# Nuclear EGFR in breast cancer suppresses NK cell recruitment and cytotoxicity

**DOI:** 10.1038/s41388-024-03211-0

**Published:** 2024-11-09

**Authors:** Angelica Escoto, Ryan Hecksel, Chance Parkinson, Sara Crane, Benjamin Atwell, Shyanne King, Daniela Ortiz Chavez, Alison Jannuzi, Barbara Sands, Benjamin G. Bitler, Todd A. Fehniger, Andrew L. Paek, Megha Padi, Joyce Schroeder

**Affiliations:** 1 University of Arizona Department of Molecular and Cellular Biology, Tucson, AZ 85721 USA; 2 University of Arizona Cancer Center, Tucson, AZ 85721 USA; 3 BIO5 Institute, Tucson, AZ 85721 USA; 4 Division of Gynecologic Oncology, Department of Obstetrics and Gynecology, University of Colorado School of Medicine, Aurora, CO 80045 USA; 5 Division of Oncology, Department of Medicine, Washington University School of Medicine, St. Louis, MO 63110 USA

**Keywords:** Breast cancer, Immunosurveillance

## Abstract

Natural Killer (NK) cells can target and destroy cancer cells, yet tumor microenvironments typically suppress NK cell recruitment and cytotoxicity. The epidermal growth factor receptor (EGFR) is a potent oncogene that can activate survival, migration, and proliferation pathways, and clinical data suggests it may also play an immunomodulating role in cancers. Recent work has demonstrated a novel role for nuclear EGFR (nEGFR) in regulating transcriptional events unique from the kinase domain. Using a novel peptide therapeutic (cSNX1.3) that inhibits retrograde trafficking of EGFR and an EGFR nuclear localization mutant, we discovered that nEGFR suppresses NK cell recruitment and cytotoxicity. RNA-Seq analysis of breast cancer cells treated with cSNX1.3 or modified to lack a nuclear localization sequence (EGFR^ΔNLS^) revealed the EGF-dependent induction of NK activating receptor ligands, while kinase inhibition by erlotinib did not impact these genes. NanoString analysis of tumor-bearing WAP-TGFα transgenic mice treated with cSNX1.3 demonstrated an increase in immune cell populations and activating genes. Additionally, immunohistochemistry confirmed an increase in NK cells upon cSNX1.3 treatment. Finally, cSNX1.3 treatment was found to enhance NK cell recruitment and cytotoxicity in vitro. Together, the data demonstrate a unique immunomodulatory role for nEGFR.

## Introduction

Natural killer (NK) cells are cytotoxic lymphocytes known to have an anti-tumor and anti-metastatic effect [1–3]. Their functions are regulated by activating (NKp46, NKp44, NKp30, NKp80, NKG2D CD16, 2B4, DNAM1) [3] and inhibitory (CD161, KLRG1, PD1, TIM3, LAG3, CD96, TIGIT) [2] receptors that regulate tumor recognition and cytotoxic activity. Current NK cell-based therapies focus on enhancing NK cell potency and NK cell specificity against the tumor cells [1, 4, 5]. However, cancer cells are efficient at evading the immune system and altering the tumor immune microenvironment in a way that can impair the recruitment and function of NK cells [6, 7]. This includes the suppression of cytokine expression (IL-2, IL-12, IL-15, IL-18) [7] and shedding of ligands (MICA, MICB, ULBPs, B7-H6) [2], a process by which enzymes known as “sheddases” cut and release the extracellular domains of integral membrane proteins [8]. These alterations in cytokine expression and shedding of ligands contribute to diminished NK cell anti-tumor activity. This type of immune suppression can also be driven by oncogenes such as the epidermal growth factor receptor (EGFR) [9].

In breast cancer, EGFR is overexpressed [10, 11] and can retro-translocate to the nucleus [12–17], potentially contributing to the underwhelming success of conventional EGFR tyrosine kinase inhibitor (TKI) and antibody-based therapies [18–22] in breast cancer. EGFR nuclear translocation is ligand-dependent and requires retrograde endosomal trafficking from the membrane to the nucleus via the endoplasmic reticulum (ER). Once trafficked to the ER, EGFR interacts with the sec61 translocon, HSP70 and importin 1β, after which it is shuttled through the nuclear pore [23]. In the nucleus, EGFR acts as a co-transcription factor and can regulate the expression of genes such as cyclin D1 and iNOS [15, 24]. Previous studies have shown that when EGFR retro-translocation is targeted, it is more effective at inhibiting cell survival than targeting EGFR’s kinase activity with the use of tyrosine kinase inhibitors [25]. Although inhibiting nEGFR reduces cell survival and migration [24–26], little is known about how nEGFR impacts the tumor immune microenvironment in breast cancer. There are several RTKs (insulin receptor, FGFR, ErbB2, ErbB3, ErbB4) [27] that also retro-translocate [28–31] to the nucleus and are associated with immune regulation [32, 33], but it is unknown how nEGFR directly impacts immune regulation.

In the current study, we have discovered that by inhibiting the retro-translocation of EGFR, we can enhance NK cell activation via the regulation of activating NK receptor ligands. Furthermore, blocking nEGFR promotes NK cell infiltration into the tumors of an EGFR-dependent breast cancer mouse model. Importantly, this type of activated immune response is elicited when the function of EGFR in the nucleus is blocked, but not when kinase activity is blocked, indicating this to be driven by nEGFR regulation. This study is the first to demonstrate the role of nEGFR in the regulation of NK cells in the tumor microenvironment, contributing to our understanding of how nEGFR promotes the progression of breast cancer.

## Results

### Nuclear EGFR drives a unique transcriptional profile

**Fig. 1:**
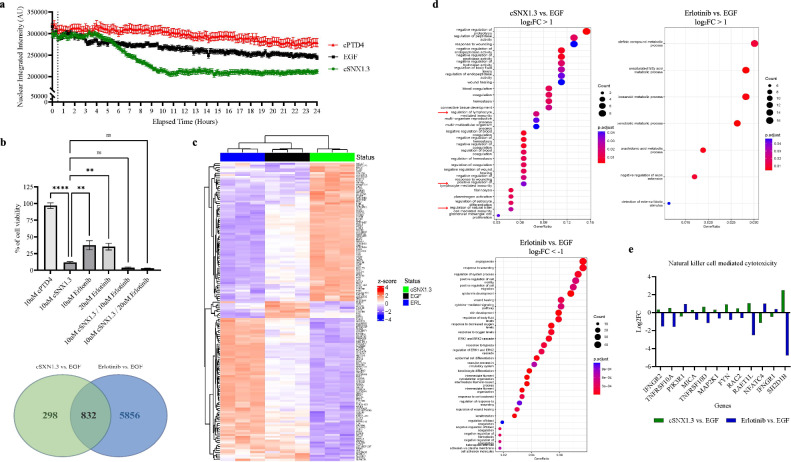
Nuclear EGFR drives a unique transcriptional profile. **a** nEGFR tracking in T47D EGFR-YFP cells. T47D cells expressing the EGFR-YFP fusion protein were serum starved overnight, then treated with 100 ng/ml of EGF, 10 μM of cSNX1.3, or 10 μM of the control peptide cPTD4 and imaged every 10 min for 24 h. Images were quantified via MATLAB. Data represents the mean ± SEM of the integrated intensity of EGFR-YFP in the nucleus of T47D cells, ±SEM. Dash line represents the time of drug added at 30 min. cSNX1.3 treatment is statistically different from cPTD4 and EGF treatments; *p* < 0.0001. Statistics were analyzed using one-way ANOVA, Dunnett’s post hoc. **b** Cell viability after cSNX1.3 and erlotinib treatment. MDA-MB-468 cells were treated with the drug treatments shown above for 72 h. Cell viability was determined with an MTT assay and normalized to vehicle control. Data represents the percentage of cell viability ± SEM of 3 biological replicates. Statistics were analyzed via one-way ANOVA, Dunnett’s post hoc; *p < 0.05, **p < 0.01, ***p < 0.001, ****p < 0.0001, compared to cSNX1.3 alone. The Bliss independence model was used to test for synergy, resulting in a score of 3.499. **c** Heatmap; MDA-MB-468 cells were serum starved for 12 h, stimulated with 10 ng/mL EGF, and treated with cSNX1.3 (10 μM) or erlotinib (15 μM) for 12 h. Samples were collected and next generation sequencing was performed by GENEWIZ from Azenta Life Sciences. Significantly differentially expressed (*P*_*adj*_ < 0.05 and abs(log_2_FC)>1) genes in the cells treated with cSNX1.3 + EGF (referred to as cSNX1.3) are visualized using their z-score across three different conditions (cSNX1.3, erlotinib, and EGF control). Venn diagram; number of significant (*P*_*adj*_ < 0.05) differentially expressed genes shared by both cSNX1.3 and erlotinib treatments. **d** Functional enrichment in the significant (*P*_*adj*_ < 0.05) differentially expressed genes between cSNX1.3 and erlotinib treatments. GO term enrichment analysis of cSNX1.3 with log_2_FC > 1 (top, left). GO term enrichment analysis of erlotinib with log_2_FC > 1 (top, right) and log_2_FC < -1 (bottom). **e** Natural killer cell-mediated cytotoxicity genes regulated by cSNX1.3 or erlotinib. Log_2_FC of significant (*P*_*adj*_ < 0.05) genes associated with natural killer cell-mediated cytotoxicity in cSXN1.3 and erlotinib. The human gene set used for identifying natural killer cell-mediated cytotoxicity genes can be found under the standard name KEGG_NATURAL_KILLER_CELL_MEDIATED_CYTOTOXICITY.

We recently reported that a peptide-based therapeutic, cSNX1.3, can block the interaction between EGFR and Sorting Nexin 1, thereby reducing the nuclear localization of EGFR [25]. Using single cell tracing of Crispr/Cas9 modified EGFR to express an EGFR-mVenus fusion protein, we validated the strong reduction of nEGFR in T47D (T47D-mVenus) breast cancer cells treated with cSNX1.3 compared to control cPTD4 treated cells (Fig. 1a). To compare the mechanism of action of cSNX1.3 with tyrosine kinase inhibition, we performed a cell survival assay on MDA-MB-468 (Fig. 1b) and T47D-mVenus (Supplementary Fig. s1a) breast cancer cells. While both cSNX1.3 and erlotinib significantly reduce cell survival, cSNX1.3 has a stronger impact – notably, combining cSNX1.3 and erlotinib had an additive effect (as determined by the Bliss independence model). We next set out to evaluate the EGF-dependent transcription profiles induced by nEGFR versus EGFR kinase-driven transcription through the inhibition of nEGFR (via cSNX1.3 treatment) or inhibition of the EGFR tyrosine kinase (via erlotinib treatment). We identified all genes that were significantly differentially expressed (*P*_*adj*_ < 0.05 and log_2_FC > 1) in the cells treated with cSNX1.3 + EGF (referred to as cSNX1.3) relative to EGF-only controls (referred to as EGF) and visualized their expression across all three conditions (Fig. 1c). Together with the Principal Component Analysis (PCA) plot of the data (Supplementary Fig. s1b), this analysis showed that cSNX1.3 and erlotinib have different effects on gene expression. There was a total of 1130 differentially expressed genes with *P*_*adj*_ < 0.05 in the cSNX1.3 vs. EGF comparison and 6688 differentially expressed genes in the erlotinib vs. EGF comparison, with the 832 genes in common showing largely opposite effects (Fig. 1c).

To help determine the biological functions perturbed by each treatment, we performed GO term enrichment analysis. Treatment with erlotinib resulted in changes in cell growth, migration, and MAP kinase activity, as expected (log_2_FC < -1). Alternatively, treatment with cSNX1.3 resulted in changes in the regulation of lymphocyte-mediated immunity and leukocyte-mediated immunity, specifically NK cell-mediated immunity, among others (log_2_FC > 1; Fig. 1d, arrows). GO term analysis did not uncover any statistically significant functional associations among the genes with log_2_FC < -1 in the cSNX1.3 treatment. While 832 genes are common between cSNX1.3 and erlotinib, those that are involved in NK cell-mediated cytotoxicity and function were downregulated by erlotinib as opposed to upregulated by cSNX1.3 (Fig. 1e). Additionally, we verified that cSNX1.3 increased expression of NKG2D ligands RAET1H, RAET1G, and RAET1L by RT-PCR and qPCR (RAET1L) (Supplementary Fig. s1c, Fig. s1d). The upregulation of these genes upon blocking EGFR nuclear translocation indicates the potential role of nEGFR in regulating the immune system, specifically in inhibiting the function of NK cells.

### Directly targeting the NLS of EGFR impacts the expression of NK cell activation genes

**Fig. 2:**
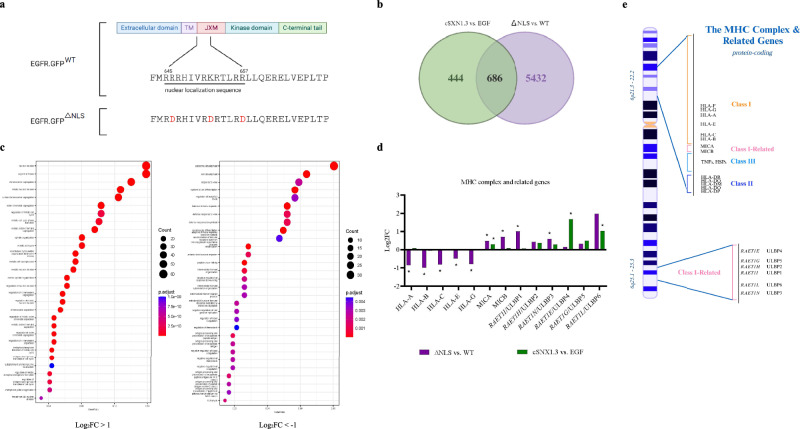
Directly targeting the NLS of EGFR alters the expression of NK cell function genes. **a** Diagram depicting the NLS in wildtype EGFR (WT; top) and EGFR mutant (ΔNLS; bottom). Three amino acids (two arginines and one lysine) in the tripartite sequence (645–657) were changed to aspartic acids (646 R > D; 653 K > D; 657 R > D). **b** Venn diagram; significantly differentially expressed (*P*_*adj*_ < 0.05) genes shared by both cSNX1.3 and ΔNLS (ΔNLS relative to its WT control). **c** Functional enrichment in the significantly (*P*_*adj*_ < 0.05) differentially expressed genes of the ΔNLS group. GO term enrichment analysis of ΔNLS with log_2_FC > 1 (left) and log_2_FC < -1 (right). **d** Natural killer cell-mediated cytotoxicity genes regulated from directly targeting the NLS of EGFR. Log_2_FC of significantly (*P*_*adj*_ < 0.05) differentially expressed genes associated with natural killer cell-mediated cytotoxicity in both ΔNLS and cSNX1.3. The human gene set used for identifying natural killer cell-mediated cytotoxicity genes can be found under the standard name KEGG_NATURAL_KILLER_CELL_MEDIATED_CYTOTOXICITY. **e** Schematic of chromosome 6, which encodes for major histocompatibility complex genes (Class I, Class II, Class III, and Class-I related).

To further validate that the effects of cSNX1.3 treatment were specific to blocking nEGFR accumulation, we generated EGFR-GFP constructs that were either wildtype (EGFR.GFP^WT^) or harbored mutations in its NLS (EGFR.GFP^ΔNLS^). The tripartite NLS is found within the juxtamembrane domain of EGFR and is composed of three basic amino acid clusters. To generate the mutant cell line, two arginines and one lysine were mutated to aspartic acids, as was done previously [34, 35] (Fig. 2a). Endogenous EGFR expression was knocked down using a lentiviral shRNA construct targeting the 3’-UTR of EGFR (Supplementary Fig. 2), followed by transfection of either EGFR.GFP^WT^ or EGFR.GFP^ΔNLS^ into MDA-MB-468 cells. EGF treatment and bulk RNA-Seq analysis revealed a total of 6118 genes that were differentially expressed with *P*_*adj*_ < 0.05 in the ΔNLS sample vs. WT. When compared to the cSNX1.3 vs. EGF differentially expressed genes, 686 genes were found to be in common (Fig. 2b).

To determine the biological functions associated with nEGFR accumulation, we performed GO term enrichment analysis on the differentially expressed genes. We found that directly targeting the NLS of EGFR impacts biological processes involved in cell cycle signaling and the immune response (Fig. 2c). Considering that NK cell-mediated cytotoxicity is a biological process that was impacted by treatment with cSNX1.3, we examined the change in expression of genes known to be regulators of NK cell-mediated cytotoxicity in the ΔNLS and cSNX1.3 treatment conditions. Blocking nEGFR by directly targeting its NLS resulted in the downregulation of HLA genes (HLA-A; HLA-B; HLA-C; HLA-E; HLA-G) known to be important inhibitory ligands for NK cells, and the upregulation of ULBP (ULBP1; ULBP3) and MIC (MICA; MICB) genes, ligands for the NKG2D receptor known to be involved in the detection and elimination of damaged, transformed, and pathogen-infected cells [36]. Similarly, treatment with cSNX1.3 impacts the regulation of MICA and the ULBP (ULBP4; ULBP6) genes (Fig. 2d). These MHC complex and related genes are all part of chromosome 6 (Fig. 2e). Overall, the results suggest that nEGFR impacts recognition of tumor cells by NK cells through the modulation of both activating and inhibitory NK receptor ligands.

### Targeting nEGFR results in modulation of the tumor immune microenvironment

**Fig. 3:**
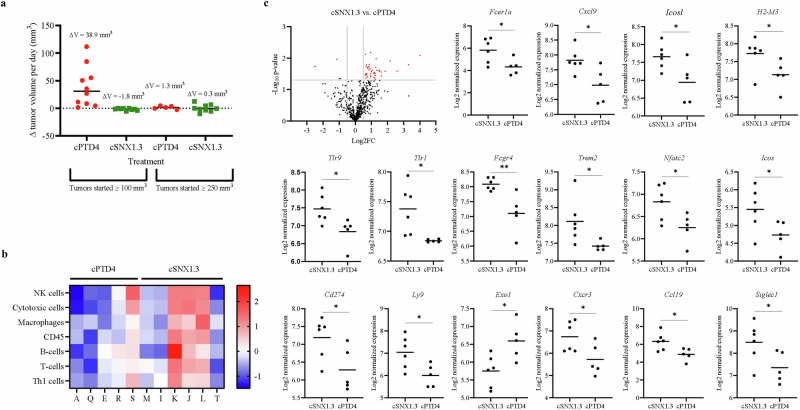
Targeting nEGFR activates the tumor immune microenvironment. ^ϯ^Tumors from the WAP-TGFα female mice treated with cSNX1.3 (*n* = 6) or cPTD4 (*n* = 5) were fixed and paraffin embedded. The embedded tissue was used for NanoString analysis. **a** Change in tumor volume per day of mice treated with cSNX1.3 or cPTD4, ±SEM. Statistics were analyzed using an unpaired *t* test, *p < 0.05, **p < 0.01, ***p < 0.001, ****p < 0.0001. **b** Heatmap demonstrating cell type score for each cSNX1.3 and cPTD4 sample. Red represents upregulation and blue represents downregulation of gene expression. **c** Volcano plot showing the differentially expressed genes in cSNX1.3 vs. cPTD4. Blue dots represent downregulated genes (4) with a log_2_FC < 0.5 and red dots represent upregulated genes (*n* = 41) with a log_2_FC > 0.5. Scatter plots showing Log2 normalized expression of genes found to be ^ϯϯ^significant (*P*_*adj*_ ≤ 0.05) between cSNX1.3 and cPTD4. ^ϯ^11 tumors that started from the ≥100 mm^3^ arm of the study (left) were used for NanoString analysis. ^ϯϯ^Unpaired *t* test; correction for multiple testing using Benjamini-Hochberg method in R.

To evaluate differential gene expression in vivo, we utilized mammary tumors generated in the WAP-TGFα transgenic mouse model [37]. Tumor-bearing females were established and then treated with cSNX1.3 or cPTD4 peptide once a tumor reached approximately 100 mm^3^ or 250 mm^3^. The mice remained in the study for 4 weeks or until they reached maximal tumor burden. Tumors grew in 1 or more of the 10 mammary fat pads. Results from the study showed an overall regression in tumor volume of mice treated with cSNX1.3 (ΔV = –1.8 mm^3^ and –3.6 mm^3^, respectively) compared to cPTD4 peptide control treatment (ΔV = 38.9 mm^3^ and 7.6 mm^3^, respectively) (Fig. 3a). The tumors that started at ≥ 100 mm^3^ had a significant change in tumor volume between cSNX1.3 and cPTD4 (*P* < 0.01). At the end of the study, mice were sacrificed and tumors were collected and paraffin embedded for gene expression profiling (NanoString). Using the nCounter mouse PanCancer IO (immune-oncology) 360 panel, the differentially expressed genes were used as marker genes to specify and quantify the different cell populations present in our samples. We used ROSALIND to perform a filtering of Cell Type Profiling, retaining all results that had scores with *P* < 0.05. The results showed a greater abundance of multiple immune cell types (NK cells, cytotoxic cells, macrophages, B cells, T cells, Th1 cells) in the tumor tissue from the mice treated with cSNX1.3 as compared to cPTD4 (Fig. 3b). Note that cytotoxic cells include both cytotoxic T cells and cytotoxic NK cells with marker genes – *Prf1*, *Gzma*, *Gzmb*, *Nkg7*, *Klrd1*, *Klrk1*, *Klrb1*, *Gzma*, NanoString Codeset: NS_*Gzmb*, *Nkg7*, *Klrd1*, *Klrk1*, *Klrb1*, *Ctsw* – as annotated in the NanoString Codeset: NS_Mm_IO_360_v1.0.

Of the 45 genes that were discovered to be differentially expressed, 16 genes were statistically significant (*P*_*adj*._ < 0.05) when cSNX1.3 and cPTD4 treatments were compared. Specifically, one gene was found to be downregulated in response to cSNX1.3 treatment that is involved in DNA repair (*Exo1*) and 15 genes were upregulated in response to cSNX1.3 treatment that are involved in immunity (Fig. 3c). The gene ontology attributes corresponding to these genes are associated with immune response-activating signal transduction, leukocyte activation, and regulation of cytokine production (Supplementary Table 1).

### Blocking nEGFR enhances NK cell recruitment and cytotoxicity

**Fig. 4:**
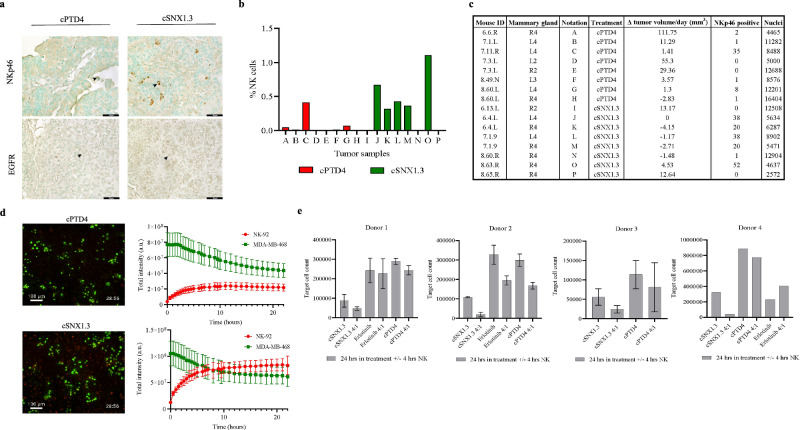
Blocking nEGFR enhances NK cell recruitment and cytotoxicity. **a** Tumors from WAP-TGFα female mice treated with cSNX1.3 (*n* = 9) or cPTD4 (*n* = 9) were fixed in 10% buffered formalin and paraffin embedded (tumors from both ≥100 mm^3^ and ≥250 mm^3^ groups were used for IHC). Slides were incubated with NKp46 primary antibody for NK cell detection, an anti-EGFR antibody, and counterstained with methyl green (nuclei). Black arrow heads indicate examples of positive staining for each. ×40 magnification for all images. **b** Quantification; identification of methyl green and positive NKp46 stain. Bar graph showing NK cells/(NK cells + nuclei) for each experimental group. **c** Table including the mice used for IHC, alongside their average tumor growth rate and the number of cells positive for NKp46 stain and methyl green (nuclei). **d** MDA-MB-468 cells were labeled with DiO, stimulated with EGF (10 ng/ml), treated with cSNX1.3 (10 μM) or cPTD4 (10 μM), and co-cultured with NK-92 cells labeled with DiD. Cells were tracked for 24 h and total intensity of both DiO and DiD staining is shown. **e** Target cell count after drug treatment and primary NK cell co-culture. MDA-MB-468 target cells were seeded at a density of 50,000 cells/well (Donors 1–3) or 400,000 cells/well (Donor 4). Target cells were stimulated with EGF (10 ng/ml) and treated with either cSNX1.3 (10 μM), erlotinib (15 μM), or cPTD4 (10 μM) for 24 h, and pre-activated primary NK cells were co-cultured with the target cells at an E:T ratio of 4:1 for 4 h.

Considering the alteration of NK cell activation and recruitment genes by RNA-Seq in vitro and NanoString analysis in WAP-TGFα tumors in vivo, we next evaluated the impact of cSNX1.3 treatment on NK cell recruitment and activation directly. We first evaluated how cSNX1.3 treatment altered the recruitment of NK cells in the WAP-TGFα model by immunohistochemistry (IHC). To address the presence of NK cells in the tumor microenvironment, we analyzed tumors derived from cSNX1.3 (*n* = 9) and cPTD4 (*n* = 9) treated mice with for NK cell infiltration and EGFR expression (Fig. 4a). While NK cells were observed in almost all cSNX1.3-treated samples, they were rarely observed in cPTD4 controls. To quantify NK cell infiltration, total nuclei and NKp46 positive cells were counted using image segmentation of 50 stitched images per slide and quantified with MatLab (Fig. 4b and Supplementary Fig. 4). Percent of NK cells per sample was determined by calculating the number of NKp46 cells/(NKp46 cells + nuclei). Next, we evaluated all individual tumors to compare their growth rate to the infiltration of NK cells observed (Fig. 4c). We found a striking concordance between NK cell infiltration and low to negative growth rates compared to no NK cell infiltration and high growth rates. Overall, our results show that cSNX1.3 treatment results in increased NK cell recruitment associated with low to negative tumor growth in WAP-TGFα transgenic tumors.

To determine how cSNX1.3 impacts the recruitment of human NK cells in vitro, MDA-MB-468 cells were labeled with DiO (Fig. 4d, green), stimulated with EGF, treated with cSNX1.3 or cPTD4, and co-cultured with NK-92 cells labeled with DiD (Fig. 4d, red). Live-cell imaging was performed and total intensity of DiO and DiD was tracked over a period of 24 h. In cells treated with cSNX1.3, we observed the NK-92 cells that were originally in suspension being recruited to the adhered tumor cells over the 24-h period, a phenomenon we did not observe with the cells treated with cPTD4 (3-fold increase in NK-92 cell recruitment compared to cPTD4) (Fig. 4d).

To determine if cSNX1.3 also results in the induction of NK cell-mediated cytotoxicity, we next evaluated cSNX1.3-dependent NK cell killing against target cells in vitro. MDA-MB-468 target cells were stimulated with EGF (10 ng/ml) and treated with cSNX1.3 (10 μM), cPTD4 (10 μM), or erlotinib (15 μM) for 24 h and then co-cultured with primary human donor NK effector cells at a 4:1 E:T ratio for an additional 4 h. Compared to cSNX1.3 alone, cells treated with cSNX1.3 and exposed to NK cells had at least a 50% decrease in viable cells compared to either cSNX1.3 alone, cPTD4, or erlotinib (Fig. 4e; note cells were limited from donor 3 and the erlotinib control was not performed). While there is significant sample-to-sample variability due to the use of primary human donor NK cells, each sample did respond to cSNX1.3 treatment with at least a 50% tumor cell killing.

## Discussion

In the present report, we have identified a novel function of nEGFR in regulating NK cell recruitment and cytotoxicity. By utilizing a targeted therapeutic, cSNX1.3, and a nEGFR mutant cell line, we found a significant difference in nEGFR and EGFR tyrosine kinase-driven gene transcription. Importantly, we found that several genes encoding NK cell activating ligands were specifically altered by nEGFR. We show that NK cells are preferentially recruited to tumors and cells treated with cSNX1.3 and that cSNX1.3 treatment also results in enhanced NK cell killing of EGFR-expressing breast cancer cells. Together, these data demonstrate a novel role for nEGFR in regulating NK cells.

While we find that erlotinib treatment impacts gene pathways long associated with EGFR function (i.e. MAP kinase pathways), blocking nEGFR transcription – either via cSNX1.3 treatment or genetically ablating the nuclear localization sequence—does not impact these canonical signaling pathways. In fact, we observe a strikingly different set of transcriptional targets from nEGFR, specifically those genes that regulate NK cells. Despite other studies showing that cancers with mutated EGFR [38, 39] treated with erlotinib leads to increased tumor killing by NK cells [9], our studies do not show a reliable increase in NK cell killing by erlotinib, especially when compared to the NK cell killing induced by cSNX1.3.

The cSNX1.3 peptide interacts with a sequence on EGFR that has significant homology with the other erbB receptors (including erbB2, erbB3 and erbB4), and erbB3 and erbB4 are expressed and functional in these cells (Supplementary Fig. 5) [40, 41]. Furthermore, the sequence of cSNX1.3 has significant homology between Snx1 and Snx2 (Supplementary Fig. 5). Therefore it is probable that cSNX1.3 impacts additional protein-protein interactions and represents a strong rationale for comparing transcriptional effects to a genetic mutation such as the ΔNLS. The ΔNLS cell line was created through the knockdown of endogenous EGFR and re-expression of the EGFR-GFP plasmids (both wildtype and mutant; Supplementary Fig. 2). Since the erbB receptors work through homo- and hetero-dimerization (and as noted above erbB3 and erbB4 are expressed in these cells), this approach also potentially represents non-EGFR-dependent transcriptional effects. Together, these factors allow for non-overlapping impacts of the two approaches. Yet, both approaches found alterations in NK cell gene expression, specifically the ULBP genes. This striking overlap (and difference from erlotinib treatment) led us to focus on the potential impact of nEGFR in NK cell recruitment and activation. Evaluation of the NanoString gene expression profiling and RNA-seq demonstrate additional cell type involvement in the response to cSNX1.3 loss of nuclear EGFR (Fig.1 and Fig. 3). Specifically, B and T lymphocytes and macrophages are found to be increased by gene expression profiling (Fig. 3). As the innate and adaptive cells of the immune system work in synergy, it is probable that there is crosstalk between the arms. For example, alterations to NK cell recruitment and activation can alter the overall inflammatory nature and induce a recruitment of T lymphocytes [42]. Alternatively, it is possible that cSNX1.3 is directly impacting macrophage activity, as EGFR is expressed and functional in these cells in regulating macrophage polarity [43]. While it will be interesting to investigate the role of nuclear EGFR in these additional immune cell types, the current in vitro data demonstrates that nuclear EGFR can directly impact the expression of NK cell activation antigens in tumor cells. The in vitro killing assays demonstrate that NK cells can induce tumor cell killing of cSNX1.3-treated tumor cells in the absence of additional immune mediators. Together, these data show a direct role for tumor cell nuclear EGFR in modulating NK cell activity, although other impacts on the immune system are likely.

Overall, NK cells are an important component of the anti-tumor immune response, capable of controlling progression and metastasis [4, 12, 44, 45]. However, breast cancer cells have developed mechanisms of escape from NK cell-mediated immunity by impairing their recruitment and cytotoxic functions. Although attempts at activating the tumor immune microenvironment have been made using antibodies and TKIs, the impact of nEGFR on immunosuppression has yet to be explored. We now show that nEGFR can regulate NK cell recruitment and cytotoxicity in EGFR-dependent breast cancer. Our findings suggest a suppressive role of nEGFR in the immune system process, specifically in NK cell function. Several avenues remain to be explored, including opportunities to better understand the mechanism by which nEGFR impacts NK cell recruitment. Insights gained from our study may help direct the development of combinatorial therapies, utilizing nEGFR inhibitors with immunotherapies.

## Materials and methods

### Cell lines

#### MDA-MB-468, NK-92, and primary NK cell lines

MDA-MB-468, T47D and NK-92 cell lines are from ATCC (and maintained according to ATCC guidelines). Leukoreduction chambers were obtained from anonymous healthy platelet donors and NK cells were purified (>95% CD56 + CD3-) using RosetteSep (Stem Cell Technologies) negative selection and Ficoll centrifugation, as described [46]. Primary NK cells were grown in RPMI-1640 medium supplemented with 10% heat inactivated human AB serum, 2 mM glutamine, 1% penicillin-streptomycin, 100 mM HEPES, 1x nonessential amino acids, and maintained in 1 ng/mL of IL-15 (Miltenyi Biotech). Cell lines are tested for mycoplasma every 6 months.

#### EGFR^WT^ and EGFR^ΔNLS^ cell lines in MDA-MB-468 cells

EGFR-GFP plasmid (a gift from Graham Carpenter) was subjected to mutagenesis using the Q5 Site-Directed Mutagenesis Kit (New England BioLabs). EGFR knockdown was created using lentiviral particles containing isopropyl-β-D-1-thiogalactopyranoside (IPTG)-inducible short hairpin RNA (shRNA) sequences and puromycin resistance (MilliporeSigma). EGFR knockdown: Construct A (5ʹ-CCGGAGAATGTGGAATACCTAAGGCTCGAGCCTTAGGTATTCCACATTCTCTTTTTG 3ʹ) Construct B (5ʹ- CCGGGCTGCTCTGAAATCTCCTTTACTCGAGTAAAGGAGATTTCAGA GCAGCTTTTTG-3ʹ). Nonspecific shRNA: Construct C (5ʹ-GCGCGATAGCGCTAATAATTT 3ʹ. Cells were incubated with hexadimethrine bromide (8 μg/mL) to increase transduction efficiency before viral particles were added at an MOI of 1. Transduced cells were then incubated with 1 mM IPTG for 2 days to establish knockdown and then remained in IPTG for the duration of the experiment.

### Mouse studies

All animal experiments were performed with approval by the University of Arizona Institutional Animal Care and Use Committee. WAP-TGFα transgenic mice (The Jackson Laboratory; Tg(WapTgfa)215Bri) were backcrossed to C57BL/6 J background. WAP-TGFα heterozygous males were bred with C57BL/6 J and offspring were genotyped using primers against the WAP-TGFα transgene. WAP-TGFα heterozygous females were entered into either the cPTD4 (control) or cSNX1.3 (treatment) groups of the study when tumors reached either 150 mm^3^ or 250 mm^3^, as indicated. Randomization was used to determine how mice were allocated to experimental groups*. Mice were added to alternating control or treatment arms as they developed tumors. Injections were administered intravenously at 10 μg/g body weight 3x/week for 4 weeks. Mice were sacrificed when tumors reached a total burden of 2000 mm^3^, when a single tumor reached a diameter of 2 cm, or after 4 weeks of drug treatment. Mice were monitored 2x/week.

*Researchers were not blinded to the experimental arm allocation.

### Immunohistochemistry (IHC) of mouse tumor tissues

Tumors were fixed in 10% neutral buffered formalin and paraffin embedded. Tissues were sectioned by the Tissue Acquisition and Cellular/Molecular Analysis Core at the University of Arizona Cancer Center. Anti-NK cell antibody NKp46 (cat. #PA5-102860; ThermoFisher Scientific) and anti-EGFR antibody EP38Y (ab52894; Abcam) were used to assess NK cells and total EGFR. Deparaffinization, rehydration, antigen retrieval and primary and secondary antibody reactions were performed according to the antibody’s manufacture specifications.

### Immunohistochemistry image segmentation and quantification

A custom MATLAB script was used to *segment IHC images and quantify methyl green and NKp46 counterstained cells. Briefly, RGB images were split into individual channels and Cellpose [47] was used to identify methyl green (channel 1) positive cells. The “nuclei” Cellpose model was used. Parameters were optimized to limit false positives for the identification of methyl green counterstained objects. Key parameters are as follows. Methyl green: (ImageCellDiameter = 40, CellThreshold = -0.5, and FlowErrorThreshold = 0.75).

For NKp46 counterstained cells, channel 2 of the original RGB images was used. A custom MATLAB script was used for image processing. Cellpose was not used for the identification of NKp46 counterstained objects as many false positives were present. Instead, background subtraction followed by Gaussian smoothing was performed. Otsu’s method was then used to automate image thresholding and create a binary image for segmentation. Finally, segmented objects were filtered based on circularity and size. Examples of segmented images are in Supplementary Fig. 4. All custom written scripts described are available upon request.

*Researchers were blinded to treatment groups to avoid bias.

### NK cell killing assay

MDA-MB-468 target cells were seeded at a density of 50,000 cells/well in a 24-well plate or 400,000 cells/well in a 6-well plate and allowed to adhere for 24 h. The target cells were then stimulated with EGF (10 ng/ml) and treated with either cSNX1.3 (10 μM) or cPTD4 (10 μM) for an additional 24 h. Prior to being co-cultured with the target cells, primary NK effector cells were pre-activated with IL-15 (50 ng/ml) for 2 h, washed, and resuspended in 1 ng/ml of IL-15 for the remainder of the assay. After 24 h of drug treatment, the pre-activated primary NK cells were co-cultured with the target cells at an effector:target (E:T) ratio of 4:1 for 4 h. After 4 h, NK cells were washed off with PBS and the remaining target cells were trypsinized and counted.

### Cell viability

MDA-MB-468 cells were treated with the indicated drugs and incubated for 72 h. The drug-containing media was decanted from the plate and replaced with media containing 250 μg/mL of 3-(4,5-dimethylthiazol-2-yl)-2,5-diphenyltetrazolium (MTT) for 80 min. The resulting formazan crystals were dissolved in 100% DMSO and the absorbance at 540 nm was detected using a Biotech Synergy LX plate reader. To test for synergy, the Bliss independence model was used. A score of < –10 indicates the interaction between two drugs is likely to be antagonistic, a score between -10 and 10 indicates the interaction is likely to be additive, and a score >10 indicates the interaction is likely to be synergistic.

### Live-cell nuclear EGFR tracking

T47D cells were modified with the eFlut plasmid system [48] to stably express mVenus [49] fused to the endogenous EGFR gene using recombinant Cas9 (Alt-R™ S.p. Cas9 Nuclease V3, IDT). T47D EGFR-mVenus cells were serum starved overnight and incubated with SPY650-DNA Probe (Spirochrome), treated with the indicated drugs and imaged every 10 min for 24 h using a Nikon Eclipse Ti microscope. Cells were imaged with the ET-EYFP and Filter Set for 800 ms (Chroma). The SPY650 DNA dye was imaged using the ET-Cy5 Filter Set for 20 ms (Chroma). Images were acquired with the Hamamatsu ORCA-Flash 4.0 camera and quantified via MATLAB.

### CELLCYTE real-time live imaging

MDA-MB-468 target cells were seeded at a density of 300,000 cells/well in a 6-well plate and labeled with Vybrant^TM^ DiO NK-92 and effector cells were labeled with Vybrant^TM^ DiD and co-cultured with the target cells along with drug treatment at an E:T ratio of 2:1. EGF (10 ng/ml) and cSNX1.3 (10 μM) or cPTD4 (10 μM) were added to the media and all cells were tracked for 24 h using the CELLCYTE X^TM^ Live Cell Imager and Analyzer.

### Bioinformatics analysis

Quality of raw reads was assessed using FastQC, and reads were trimmed using TrimGalore 0.6.7 with a Phred score quality threshold of 20 (the default) and removing Illumina adapters. Trimmed reads were then aligned to the GRCh38 reference human genome using STAR 2.7.9a with default parameters. Aligned reads were then quantified using featureCounts from the Rsubread package, along with the GRCh38 gtf file, and specifying that the sequencing was paired end but not strand specific.

The R package DESeq2 was used to test for differential expression between the different group comparisons and to select all genes with a *P*_*adj*_ value < 0.05. The R package clusterProfiler was used to evaluate functional enrichment in the differentially expressed genes between the different groups. We ran overrepresentation analysis on Gene Ontology using the inputs: differentially expressed genes, the universe, the species-specific annotation database (org.Hs.eg.db), the BP ontology, a *p* value adjustment method (Benjamini-Hochberg), and *p* value and q-value cutoffs (0.05). The R package ComplexHeatmap was used to create a heatmap visualizing the vst-normalized data created using the DESeq2 package.

### NanoString analysis

As described in Jordan et al. [50]. Formalin-fixed paraffin-embedded (FFPE) murine tumor specimens were sectioned and for each tumor block there were two, 20 μm sections. RNA was extracted with High Pure FFPET RNA Isolation Kit (Roche) and the RNA quantity and quality were determined with an RNA ScreenTape on a TapeStation 4150 (Agilent Technologies). RNA concentration was calculated by comparing the RNA ladder and the percentage of RNA fragments greater than 200 bp. RNA (150 ng) was combined with hybridization buffer and the reporter CodeSet for the Murine PanCancer IO 360 Panel (NanoString Technologies) and incubated for 20 h at 65 °C. The hybridized reaction was analyzed on an nCounter SPRINT Profiler (NanoString Technologies).

mRNA expression, normalization, fold changes and *p* values were calculated using ROSALIND. *Normalization*. Normalization involves dividing counts within a lane by the geometric mean of the normalizer probes from the same lane. Housekeeping probes used for normalization were selected based on the geNorm algorithm as implemented in the NormqPCR Bioconductor package in R. *Differential expression*. Differential expression is calculated based on user specified groups. Fold changes and *p* values are calculated using the method described in the nCounter Advanced Analysis 2.0 User Manual. *P* value adjustment was performed using the Benjamini-Hochberg of estimating false discovery rates. *Cell type profiler*. The method to quantify abundance of cell populations uses marker genes expressed stably and specifically in given cell types. ROSALIND filters results to include those that have scores with *p* values ≤ 0.05. Algorithm details of all processes can be found in the nCounter Advanced Analysis 2.0 User Manual.

## Supplementary information

Supplemental Material Legends

Supplementary Figure 1

Supplementary Figure 2

Supplementary Figure 4

Supplementary Figure 5

Supplementary Table 1

Supplementary Table 2

## Data Availability

RNA-Seq data is available in the Gene Expression Omnibus under accession number GSE273890.
