# Supplementary material for: Nuclear EGFR in breast cancer suppresses NK cell recruitment and cytotoxicity
Source: Oncogene. 2024 Nov 9;44(5):288–95. doi: 10.1038/s41388-024-03211-0 (PMC11779631; doi:10.1038/s41388-024-03211-0)
Supplement: Supplementary file 1 — Supplemental Material Legends [file 41388_2024_3211_MOESM1_ESM.pdf]

Supplementary Figure 1

s1a. cSNX1.3 reduces cell viability in T47D EGFR-mVenus cells. T47D cells expressing the EGFR-mVenus fusion protein were treated with dose response of cPTD4, cSNX1.3, or sapitinib as shown above. Cell viability was determined by MTT assay after 72 hours of drug treatment. Data represents the mean ± SEM of the percentage of cell viability, normalized to drug vehicle treatment. Statistical significance was determined by comparing cSNX1.3 or sapitinib vs the same concentration of cPTD4 using Dunnett's multiple comparisons test, * $p < .05$, *** $p < .0005$, **** $p < .0001$

s1b. Principal Component Analysis of all samples profiled by RNA-Seq: MDA-MB-468 cells treated with EGF only (EGF), EGF + erlotinib (ERL), or EGF + cSNX1.3 (cSNX1.3), and MDA-MB-468 cells with endogenous EGFR knocked down and transfected with wildtype EGFR (WT) or EGFR mutated in its NLS (NLS).

s1c. cSNX1.3 induces expression of RAET1H, RAET1G, and RAET1L. MDA-MB-468 cells were treated with 10 ng/mL of EGF (10 ng/mL) and 10 μM cPTD4 or cSNX1.3 for 12 hours and analyzed by RT-PCR for the indicated genes.

s1d. cSNX1.3 induces the expression of RAET1L at varying concentrations. MDA-MB-468 cells were treated with 10 ng/mL of EGF (10 ng/mL) and 0.1 μM, 1 μM, 5 μM, or 10 μM cPTD4 or cSNX1.3 for 12 hours and analyzed by qPCR. Expression normalized to GAPDH expression and fold change determined by comparing each treatment (cSNX1.3) with its control (cPTD4). Error bars demonstrate standard deviation.

Supplementary Figure 2

Endogenous EGFR knockdown using inducible shRNA. MDA-MB-468 cells were transduced with lentiviral vectors containing inducible shRNA to knockdown endogenous EGFR. Cells were treated with 1 mM IPTG in media for 48 hours to induce shRNA activity before immunoblotting with anti-EGFR antibody (top). Nuclear localization of EGFR wildtype tagged with GFP ($EGFR.GFP^{WT}$) compared to EGFR NLS mutant tagged with GFP ($EGFR.GFP^{\Delta NLS}$). The nuclear stain, Hoescht 33342, was used to identify the localization of EGFR relative to the nucleus (bottom).

Supplementary Table 1

Gene ontology attributes corresponding to the 16 genes significantly ($P_{adj.} < 0.05$) differentially expressed between cSNX1.3 and cPTD4.

Supplementary Figure 4

Tumors from WAP-TGFα female mice (from both arms of the mice study ($\geq 100\ mm^3$ and $\geq 250\ mm^3$)) treated with cSNX1.3 or cPTD4 and stained with NKp46 or EGFR primary antibody overnight. Negative control (rabbit IgG isotype control) shown. Examples of image segmentation shown. 20X magnification for NKp46 staining of stitched images and 40X magnification for EGFR staining of single images.

Supplementary Figure 5

cSNX1.3 interacts with a sequence on EGFR that has > 80% homology with other erbB receptors.
