# Supplementary figures and images for: Nuclear EGFR in breast cancer suppresses NK cell recruitment and cytotoxicity
Source: Oncogene. 2024 Nov 9;44(5):288–95. doi: 10.1038/s41388-024-03211-0 (PMC11779631; doi:10.1038/s41388-024-03211-0)

s1a

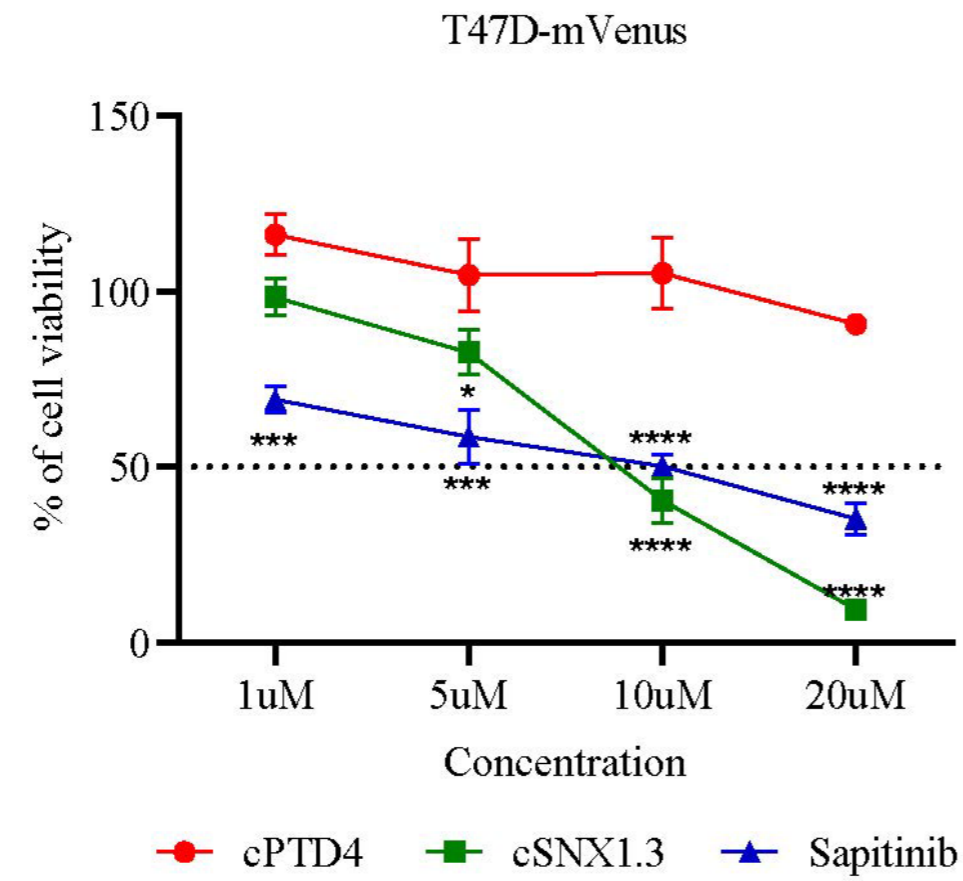

s1b

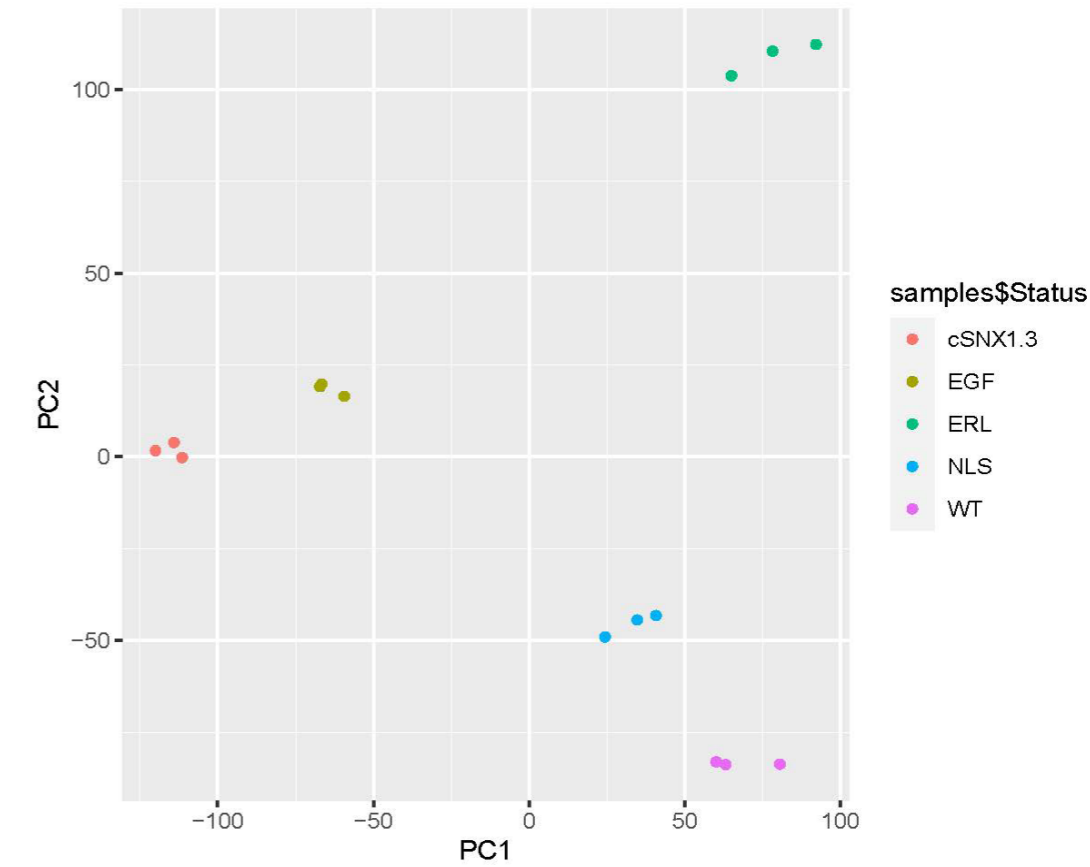

s1c

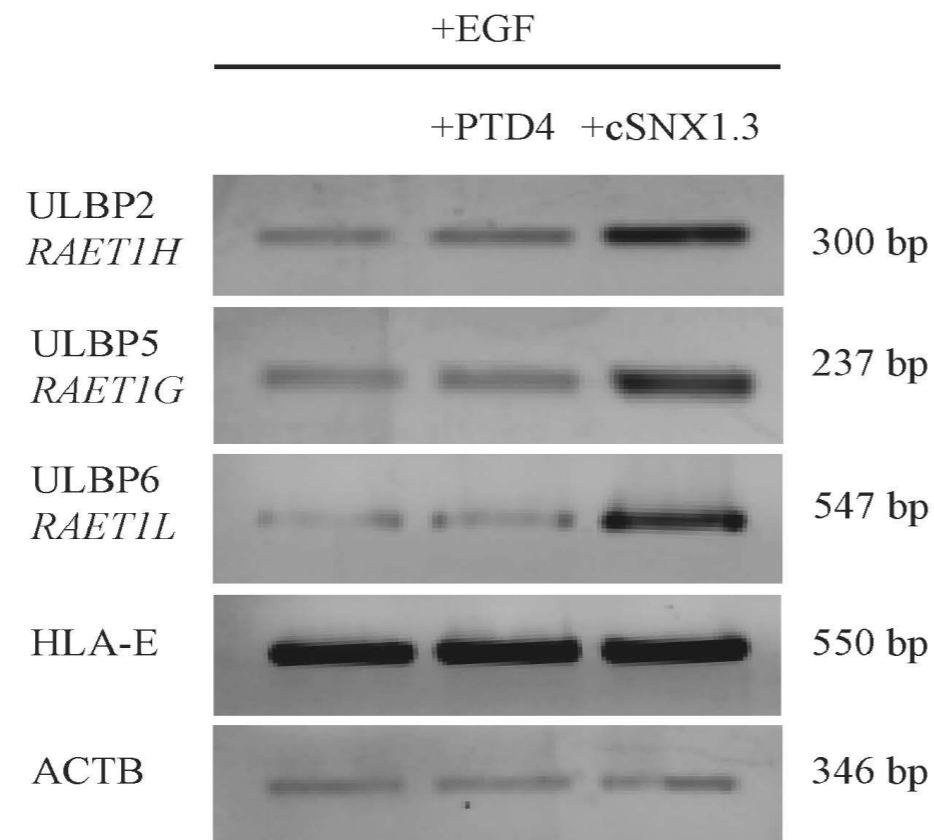

s1d

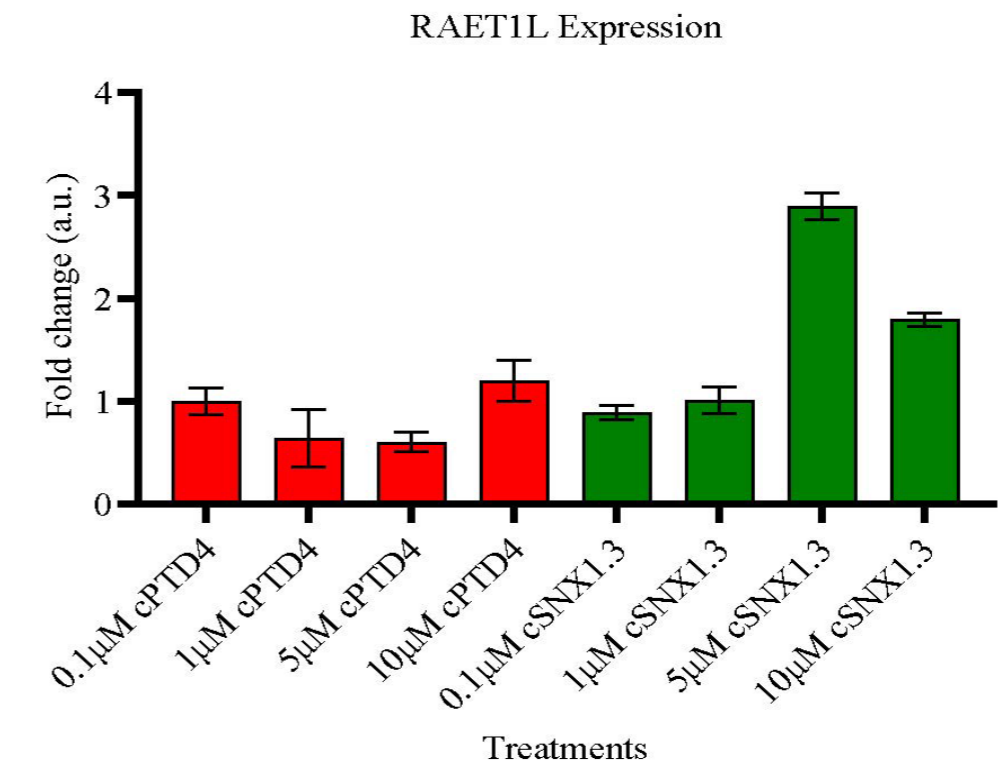

Supplement: Supplementary file 2 — Supplementary Figure 1 [file 41388_2024_3211_MOESM2_ESM.pdf]

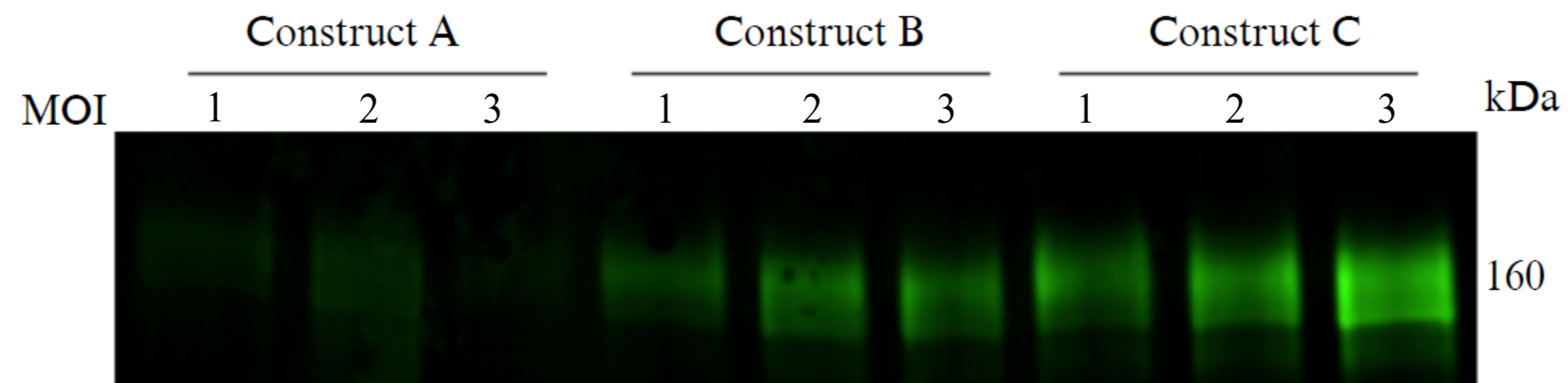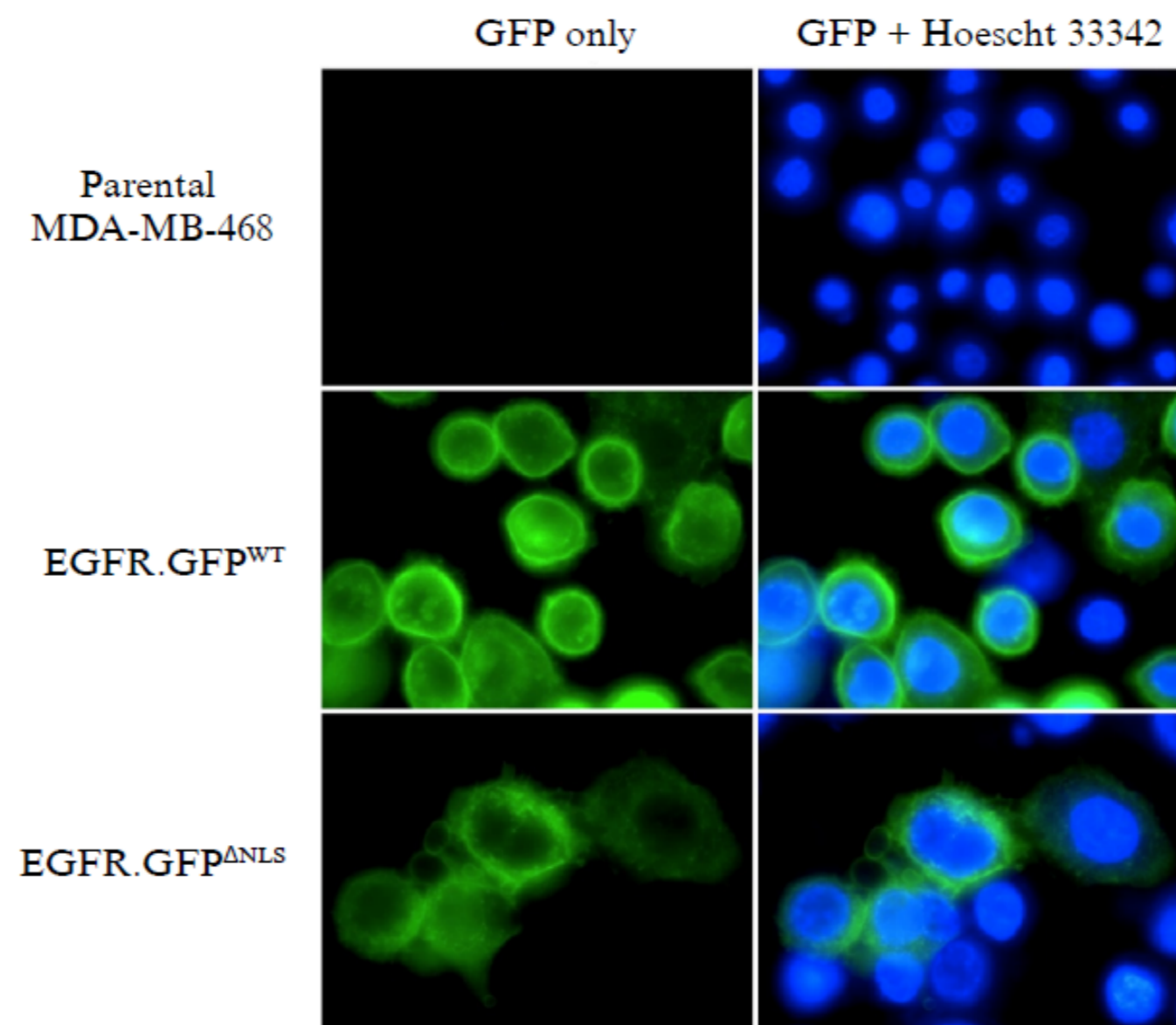

Supplement: Supplementary file 3 — Supplementary Figure 2 [file 41388_2024_3211_MOESM3_ESM.pdf]
