# Supplementary material for: Nuclear EGFR in breast cancer suppresses NK cell recruitment and cytotoxicity
Source: Oncogene. 2024 Nov 9;44(5):288–95. doi: 10.1038/s41388-024-03211-0 (PMC11779631; doi:10.1038/s41388-024-03211-0)
Supplement: Supplementary file 4 — Supplementary Figure 4 [file 41388_2024_3211_MOESM4_ESM.pdf]

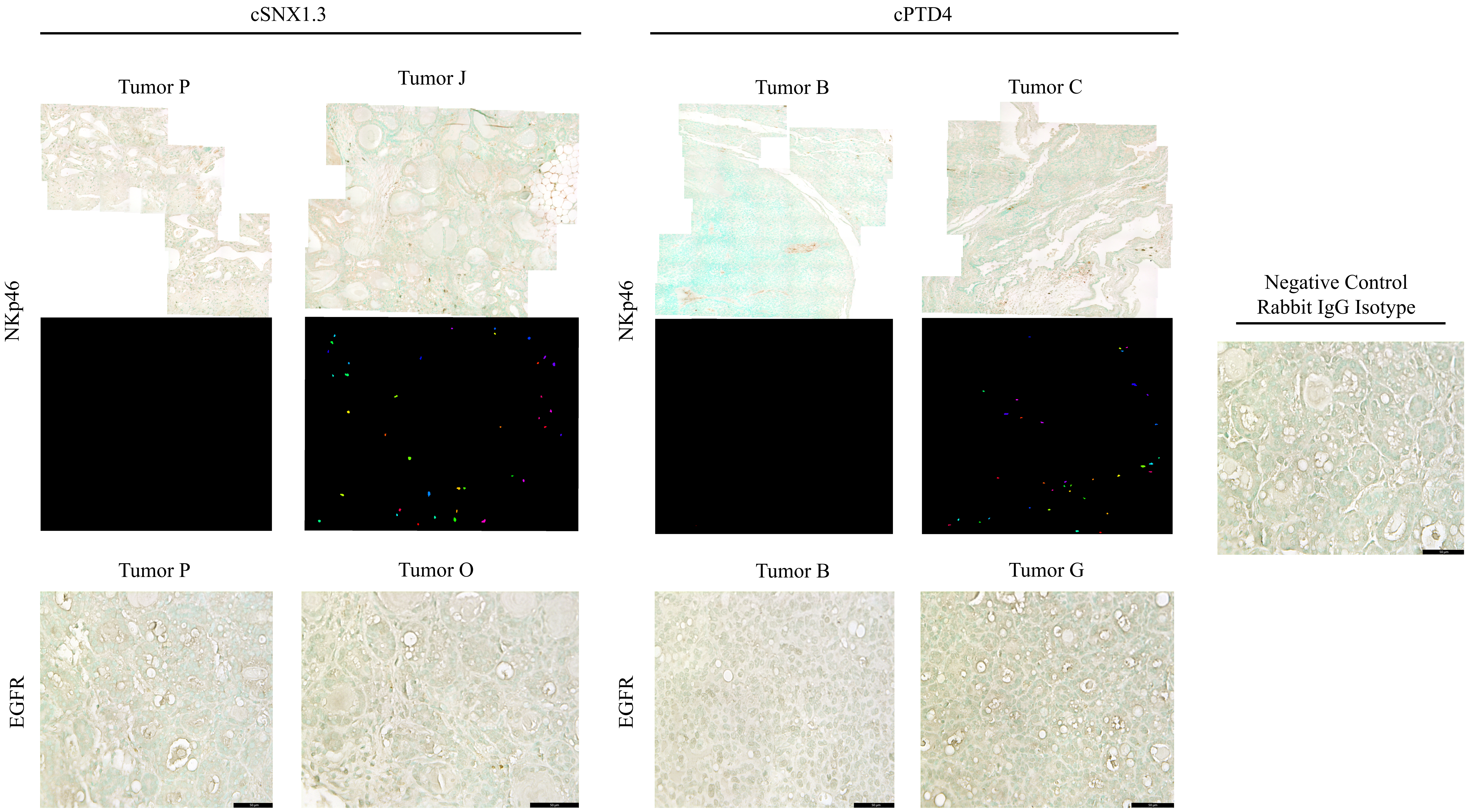
cSNX1.3
cPTD4
Tumor P
Tumor J
Tumor B
Tumor C
NKp46
NKp46
Negative Control
Rabbit IgG Isotype
Tumor P
Tumor O
Tumor B
Tumor G
EGFR
EGFR
