# Supplementary material for: Nuclear EGFR in breast cancer suppresses NK cell recruitment and cytotoxicity
Source: Oncogene. 2024 Nov 9;44(5):288–95. doi: 10.1038/s41388-024-03211-0 (PMC11779631; doi:10.1038/s41388-024-03211-0)
Supplement: Supplementary file 5 — Supplementary Figure 5 [file 41388_2024_3211_MOESM5_ESM.pdf]

		% Identity	E Value
EGFRSBD	VYMIMVKCWMIDADSRPKFRELIIEFSKMARDPQRY	Query	
EGFR	VYMIMVKCWMIDADSRPKFRELIIEFSKMARDPQRY	100%	$2e^{-23}$
ErbB2	VYMIMVKCWMIDSECRPRFRELVSEFSRMARDPQRF	77.87%	$4e^{-30}$
ErbB3	VYMVMVKCWMIDENIRPTFKELANEFTRMARDPPRY	69.44%	$2e^{-24}$
ErbB4	VYMVMVKCWMIDADSRPKFKELAAEFSRMARDPQRY	86.11%	$3e^{20}$

		% Identity	E Value
cSNX1.3	KNHVIKYLETLLYSQQQLAKYWEAFL	Query	
SNX1	KNHVIKYLETLLYSQQQLAKYWEAFL	100%	$4e^{-27}$
SNX2	KTVIIKYLESLVQTQQQLIKYWEAFL	88%	$1e^{-14}$

Residue	Color	Property
AVFPMILW	RED	Small (small+ hydrophobic)
DE	BLUE	Acidic
RK	MAGENTA	Basic - H
STYHCNGQ	GREEN	Hydroxyl + sulfhydryl + amine + G
Others	Grey	Unusual amino/imino acids etc.
